# Milton’s Domestic Habits

**Published:** 1864-12

**Authors:** 


### MILTON'S DOMESTIC HABITS.

BY MR. KEIGHLY.

AT his meals he never took much wine or any other fermented liquor, and he was not fastidious in his food; yet his taste seems to have been delicate and refined, like his other senses, and he had a preference for such viands as were of an agreeable flavor. In his early years he used to sit up late at his studies, and perhaps he continued this practice while his sight was good; but in his latter years he retired every night at 9 o'clock, and lay till 4 in summer, and 5 in winter, and, if not disposed then to rise, he had some one to sit at his bedside and read to him. When he rose he had a chapter of the Hebrew Bible read to him, and then, with of course the intervention of breakfast, studied till 12. He then dined, took some exercise for an hour—generally in a chair, in which he used to swing himself—and afterward played on the organ or the bass-viol, and either sang himself or made his wife sing, who, as he said, had a good voice but no ear. He then resumed his studies till 6, from which hour till 8 he conversed with those who came to visit him. He finally took a light supper, smoked a pipe of tobacco, and drank a glass of water, after which he retired to rest. . . . Like many other poets, Milton found the stillness, warmth, and recumbency of bed favorable to composition; and his wife said that before rising of a morning he often dictated to her twenty or thirty verses. A favorite position of his when dictating his verses, we are told, was that of sitting with one of his legs over an arm of his chair. His wife related that he used to compose chiefly in the winter, which account is confirmed by the following passage in his life by Phillips: "There is a remarkable passage in the composition of Paradise Lost which I have a particular occasion to remember: for, whereas I had the perusal of it from the very beginning, for some weeks, as I went from time to time to visit him, in a parcel of ten, twenty, or thirty verses at a time, which, being written by whatever hand came next, might possibly want correction as to the orthography and pointing; having, as the summer came on, not been shown any for a considerable while, and desiring to know the reason thereof, was answered that 'his veins never happily flowed but from the autumnal equinox to the vernal, and that whatever he attempted (at other times) was never to his satisfaction, though he courted his fancy never so much;' so that in all the years he was about this poem, he may be said to have spent but half his time therein. Milton's conversation is stated to have been of a very agreeable nature. His daughter Deborah said that he was 'delightful company, the life of the conversation, and that on account of a flow of subject, and an unaffected cheerfulness and civility.' Richardson, to whom we are indebted for the preservation of this testimony, adds that 'he had a gravity in his temper, not melancholy—or not till the latter part of his life—not sour, not morose nor ill-natured, but a certain severity of mind; a mind not condescending to little things.'"

